# Translation and cross-cultural adaptation of the Cornell Assessment of Pediatric Delirium scale for the Portuguese language

**DOI:** 10.5935/0103-507X.20180033

**Published:** 2018

**Authors:** Marina dos Santos Ramos Barbosa, Maria do Carmo Menezes Bezerra Duarte, Viviane Camila de Souza Bastos, Lívia Barboza de Andrade

**Affiliations:** 1 Instituto de Medicina Integral Professor Fernando Figueira - Recife (PE), Brasil.; 2 Hospital Esperança Recife Rede D'Or São Luiz - Recife (PE), Brasil.

**Keywords:** Delirum/diagnosis, Surveys and questionnaires, Translating, Intensive care units, neonatal

## Abstract

**Objective:**

This study sought to translate the Cornell Assessment of Pediatric Delirium from English into Brazilian Portuguese and cross-culturally adapt it for use in Brazil.

**Methods:**

Following the authorization granted by its main author, the processes of translation and cross-cultural adaptation were performed with regard to the Cornell Assessment of Pediatric Delirium in accordance with the following internationally recommended steps: translation of the original into Portuguese by two native speakers of the target language; synthesis of the translated versions; back-translation by two native speakers of the original language; review and harmonization of the back-translation; a review of the Portuguese version of the Cornell Assessment of Pediatric Delirium by an expert panel composed of specialists; pretesting including assessments of clarity, comprehensibility, and acceptability of the translated version using a sample of the target population; and finishing modifications to achieve the final version.

**Results:**

The translation and cross-cultural adaptation of the Cornell Assessment of Pediatric Delirium followed international recommendations. The linguistic and semantic issues that emerged during the process were discussed by the expert panel, which unanimously agreed to slight modifications. During pretesting, the Cornell Assessment of Pediatric Delirium was administered to 30 eligible children, twice per day; the final version was easy to understand, could be completed quickly, and showed a high inter-rater correlation coefficient (0.955).

**Conclusions:**

The translation of the Cornell Assessment of Pediatric Delirium into Brazilian Portuguese and its cross-cultural adaptation were successful and preserved the linguistic and semantic properties of the original instrument. The Cornell Assessment of Pediatric Delirium proved to be easy to understand and could be completed quickly. Additional studies are needed to test the validity and psychometric properties of this version in Brazil.

## INTRODUCTION

*Delirium* is an acute brain dysfunction that occurs among severely ill patients and is associated with longer intensive care unit (ICU) stays, higher mortality risks, and prolonged mechanical ventilation.^(^^1^^-^^3^^)^

According to the fifth edition of the Diagnostic and Statistical Manual of Mental Disorders (DSM-V),^(^^4^^)^ the criteria for *delirium* are neurological disturbances that develop over a brief period of time that tend to fluctuate in severity throughout the day, disturbance in attention, and disturbance in cognition. *Delirium* can be classified as hyperactive, hypoactive, or mixed.^(^^3^^,^^4^^)^ This condition exhibits a high prevalence among adults (50 - 80%);^(^^5^^)^ among children, it is associated with perceptual, motor, and behavioral problems, and thus might become the basis for posttraumatic stress disorder.^(^^6^^,^^7^^)^

The risk factors for *delirium* among children remain poorly understood. It is assumed they are similar to those identified for adults, the most frequent of which are pain, separation anxiety, caregiver absence, admission to the ICU, anticholinergic medications, sleep deprivation, the number of procedures, and the use of sedatives or analgesics.^(^^8^^-^^11^^)^

The prevalence and diagnosis of *delirium* within the pediatric setting have not yet been well elucidated because of the limitations of the available screening instruments and the lack of studies on this subject. This situation makes the diagnosis and interpretation of the effect of *delirium* difficult, especially in cases of young children with neurological dysfunction, whose neurocognitive function cannot be assessed.^(^^6^^,^^12^^-^^14^^)^ Several screening instruments for children admitted to pediatric ICU have been suggested in recent years, including the Pediatric Confusion Assessment Method for the Intensive Care Unit (pCAM-ICU), the Sophia Observation Withdrawal Symptoms-Pediatric Delirium scale,^(^^15^^)^ and the Cornell Assessment of Pediatric Delirium (CAPD).^(^^16^^)^

The CAPD was designed to diagnose all types of *delirium* for all pediatric age ranges and was validated in the United States. Its advantages are that it is easy to use, allows for a quick observational assessment, is able to detect all three forms of *delirium*, and can be applied by a multidisciplinary team to children of any age, regardless of the presence of developmental delays.^(^^6^^,^^16^^)^

Since 2016, the European Society of Pediatric and Neonatal Intensive Care (ESPNIC) has recommended the CAPD as the assessment to diagnose *delirium* among children and infants (grade of recommendation: A).^(^^15^^)^ A multicenter study conducted across the United States, the Netherlands, New Zealand, Australia, and Saudi Arabia investigated the prevalence of pediatric *delirium* using the CAPD and found a prevalence of 25%, with most children being under 2 years old.^(^^2^^)^ No version of the CAPD is currently available in Brazil.

For the CAPD to be used in Brazil, it must be translated into Brazilian Portuguese and cross-culturally adapted given the social and cultural characteristics as well as the ethnic diversity of this country. The translation and cross-cultural adaptation of instruments does not merely involve a simple translation of the original and a comparison to a back-translation. Although no consensus exists regarding the best strategy, it is recommended that the process consist of a combination of the literal translation of words and sentences from one language to another and a careful tuning that considers the cultural context and lifestyles of the target population of the translated version.^(^^17^^-^^22^^)^

The present study performed a translation of the CAPD into Brazilian Portuguese and a cross-cultural adaptation.

## METHODS

The present methodological study translated the CAPD into Brazilian Portuguese and cross-cultural adapted its content. The CAPD is an instrument used to diagnose *delirium* among children under intensive care.^(^^16^^)^ The present study only began after the author of the original scale, Dr. Chane Traube of Weil Cornell Medical College, New York, United States, granted us authorization. Furthermore, the Ethics Committee for research involving human participants of the *Instituto de Medicina Integral Professor Fernando Figueira* (IMIP), Recife, Pernambuco (PE), Brazil, approved this study under ruling no. 2,349,306.

The procedures adopted followed the model formulated by Reichenheim and Moraes^(^^17^^)^ and included the following steps: authorization by the CAPD's main author; translation and its equivalence; back-translation and its equivalence; analysis by an expert panel; pretesting with the target population; and a review and formulation of the final version.

### CAPD description

The CAPD is composed of eight questions to be answered based on observation. Each item is scored from 0 to 4; a total score of 9 or higher indicates *delirium*. The CAPD was internationally validated following a detailed psychometric assessment, and it has been translated and adapted for the Japanese population.^(^^16^^,^^21^^,^^22^^)^

The steps in the processes of translation and cultural adaptation followed accepted international recommendations,^(^^17^^)^ i.e., authorization for translation and cultural adaptation from the CAPD's main author; independent translation from English into Portuguese by two translators who were fluent in English; the synthesis of both versions to investigate linguistic, semantic, idiomatic, conceptual, and contextual discrepancies and obtain a single version; back-translation (the single version in Portuguese was obtained through synthesis and was back-translated into the original language of English by two native English speakers fluent in Portuguese); review and harmonization of the back-translation to obtain a single version; review by an expert panel composed of specialists with practical experience in the field of interest; pretesting to assess the clarity, comprehensibility, and acceptability of the version applied to the target population; the analysis of possible problems during application of the scale, including any necessary corrections and adjustments; and the resulting modifications and formulation of the final version.

### Translation and cross-cultural adaptation

Two experienced bilingual translators who were also native Brazilian Portuguese speakers translated the questionnaire and accompanying instructions into Portuguese. These translations were independently performed.

### Translation synthesis

The two independently translated versions were compared and analyzed. The translators and study coordinator met, and consensus was applied to solve all disagreements. The result was a single version of the scale in Portuguese.

### Back-translation into English

The single version resulting from the synthesis of the two Brazilian Portuguese versions was independently back-translated into English by two native-English-speaking translators who were fluent in Portuguese. The translators were not familiar with the notions approached in the questionnaire and had no knowledge of the original English version. Next, the five versions were reviewed and assessed by the main author of the original CAPD and by an expert panel composed of 16 specialists in the subject of interest (i.e., physical therapists, pediatric intensivists, nurses, and a neurologist).

The aim of this step was to determine the best solutions to the discrepancies and provide various alternatives for translation, thereby solving the following types of disagreement: conceptual (concerning the conceptual formulation of the assessment), idiomatic (concerning various linguistic expressions), semantic (concerning differences related to the instrument's content), and experiential (related to cultural differences).

Following the expert panel's meeting, a prefinal version of the CAPD was obtained, which was used in the pretesting step, also known as the "feasibility" step or "cognitive unfolding".^(^^17^^,^^18^^,^^23^^)^ This version was applied in the pretesting by the principal investigator and a qualified physical therapist who received standardized training on the use of the CAPD. These applications were performed independently.

The aim of this step was to detect problems during the application of the instrument and suggest solutions to improve its comprehensibility. For this purpose, the instrument was administered twice per day, in the morning and afternoon, to 30 children recruited from the IMIP pediatric ICU who used sedative-analgesic drugs for at least 48 hours, regardless of mechanical ventilation. Children with scores less than -3 on the Richmond Agitation-Sedation Scale (RASS), which is indicative of deep sedation, were excluded.^(^^13^^,^^14^^)^ Once consent was obtained, sociodemographic and specific data were collected.

The data are expressed as frequencies, means, medians, and their corresponding coefficients of dispersion. Data normality was investigated using the Kolmogorov-Smirnov test. The intraclass correlation coefficient (ICC) was calculated to assess inter-rater reliability. An ICC above 0.75 was considered as indicating good-to-excellent reliability.^(^^23^^)^

At the end of the process, each item was reviewed, and pertinent modifications suggested during the pretesting step were included, resulting in the final version of the CAPD. Thus, the final version of the CAPD adapted into Brazilian Portuguese was formulated.

## RESULTS

The first step of the translation process resulted in two Portuguese versions of the CAPD. During the synthesis step, the authors considered a combination of the two versions because the translations were similar, and the terms that differed were synonymous. Following the back-translation from Portuguese into English, no changes were made because no discrepancies were observed between the original and back-translated versions.

Table 1 describes the items assessed and modified by the CAPD's main author and the expert panel. Item #5 was considered to exhibit slight differences relative to the original because the two terms used (*agitado*, which was translates as "agitated", and *inquieto*, which translates as "restless") were considered synonyms. Although one of the eight items was considered to differ slightly from the original, the back-translation of both terms resulted in the use of the same word as in the original (i.e., "restless") during the formulation of the prefinal version and did not interfere with the semantics of the items.

**Table 1:** Description of the translated and back-translated versions assessed by an expert panel and the CAPD’s main author

Original version	Portuguese translated version #1	Portuguese translated version #2	Synthetized Portuguese version	Back-translated English version #1	Back-translated English version #2
Does the child make eye contact with the caregiver?	1. *A criança faz contato visual com cuidador?*	1. *A criança faz contato visual com cuidador?*	1. *A criança faz contato visual com cuidador?*	1. Does the child make eye contact with the caregiver?	1. Does the child make eye contact with the caregiver?
Are the child’s actions purposeful?	2. *As reações da criança são propositais?*	2. *As reações da criança são propositais?*	2. *As reações da criança são propositais?*	2. Are the child’s actions purposeful?	2. Are the child’s actions purposeful?
Is the child aware of his/her surroundings?	3. *A criança está consciente do ambiente ao seu redor?*	3. *A criança está consciente do que a cerca?*	3. *A criança está consciente do que a cerca?*	3. Is the child aware of his/her surroundings?	3. Is the child aware of his/her surroundings?
Does the child communicate needs and wants?	4. *A criança comunica necessidades e desejos?*	4.* A criança comunica necessidades e desejos?*	4. *A criança comunica necessidades e desejos?*	4. Does the child communicate needs and wants?	4. Does the child communicate needs and wants?
Is the child restless?	5. *A criança é agitada?*	5. *A criança é inquieta?*	5. *A criança é agitada?*	5. Is the child restless?	5. Is the child restless?
Is the child inconsolable?	6. *A criança é inconsolável?*	6. *A criança é inconsolável?*	6. *A criança é inconsolável?*	6. Is the child inconsolable?	6. Is the child inconsolable?
Is the child underactive – very little movement while awake?	7. *A criança está hipoativa – muito pouco movimento durante a vigília?*	7. *A criança está hipoativa – muito pouco movimento durante a vigília?*	7. *A criança está hipoativa – muito pouco movimento durante a vigília?*	7. Is the child underactive – very little movement while awake?	7. Is the child underactive – very little movement while awake?
Does it take the child a long time to respond to interactions?	8. *A criança leva muito tempo para responder às interações?*	8. *A criança leva muito tempo para responder às interações?*	8. *A criança leva muito tempo para responder às interações?*	8. Does it take the child a long time to respond to interactions?	8. Does it take the child a long time to respond to interactions?

The expert panel agreed on most of the analyzed items. However, the experts found that the Portuguese translation of one item resulted in two synonymous words that could be interpreted differently. Thus, both terms (*agitada*/*inquieta*) were included because both resulted in the same English word (i.e., "restless") on back translation.

The Portuguese version of the CAPD was used for the pretesting step. The eight items were observationally assessed, which required 2 to 5 minutes per patient at the pediatric ICU. Following pretesting, three changes were made to improve the comprehensibility of the scale. On question #2, the term *reações* ("reactions") was changed to *ações* ("actions"), and on questions #5 and #6, é was changed to *está* (in Portuguese, the verb *to be* has two forms, *ser* and *estar*; *ser* refers to something permanent, whereas *estar* refers to something transient), thereby resulting in the final version (Table 2). In the case of children under 12 months of age, the CAPD was applied with the help of a table of developmental anchor points based on the recommendation given by the main author of the original CAPD, who also sent us a copy of this table.^(^^16^^)^

**Table 2:** Final Portuguese translated version of the Cornell Assessment of Pediatric Delirium

Pontuação RASS _______________ (se -4 ou -5, não ir adiante)						
Favor responder as seguintes perguntas, com base em suas interações com o paciente durante seu plantão:						
	Nunca 4	Raramente 3	Às vezes 2	Frequentemente 1	Sempre 0	Pontuação
1. A criança faz contato visual com o cuidador?						
2. As ações da criança são propositais?						
3. A criança está consciente do que a cerca?						
4. A criança comunica necessidades e desejos?						
	Nunca 0	Raramente 1	Às vezes 2	Frequentemente 3	Sempre 4	Pontuação
5. A criança está agitada/inquieta?						
6. A criança está inconsolável?						
7. A criança está hipoativa - muito pouco movimento durante a vigília?						
8. A criança leva muito tempo para responder às interações?						
					Total:	

RASS – Richmond Agitation-Sedation Scale.

The results of the pretesting period corresponded to assessing the application of the scale to 30 children twice per day (morning and afternoon) by two examiners (the principal investigator and a duly trained collaborator). The biological and clinical data of the participants are described in table 3. The median score on the CAPD was 8 (minimum: 0; maximum: 20). The ICC between both examiners was 0.955.

**Table 3:** The biological and clinical characteristics of the 30 children recruited at the intensive care unit for the pretesting step of the cross-cultural adaptation process

Analyzed variables	Children (n = 30)
Weight (kg)	13.5 (9 - 18)
Age (months)	29 (11 - 72)
Males	18 (60)
Length of stay at ICU (days)	12 (8 - 16)
Length of stay at hospital (days)	16.5 (12 - 24)
Duration of MV (days)	8 (6 - 12)
Duration of sedation	7.5 (6 - 11)
Diagnosis	
Pneumonia	9 (30)
Sepsis	5 (16.6)
PO abdominal surgery	5 (16.6)
Heart disease	4 (13.3)
Other	7 (23.5)

ICU - intensive care unit; MV - mechanical ventilation; PO - postoperative. Results are expressed as n (%), median, and interquartile range.

## DISCUSSION

The present article describes the processes of translating the CAPD from English to Brazilian Portuguese and ensuring a cross-cultural adaptation. The steps of these processes were judiciously followed in agreement with the recommendations found in the literature. The linguistic and semantic equivalences between the original and the Brazilian Portuguese versions were satisfactory, and no divergences occurred.

The processes of translation and cross-cultural adaptation are complex and indispensable, and the characteristics of the original version must be preserved. Adaptation is important because of the heterogeneity that exists among populations, including the use of regional expressions.^(^^20^^)^

The Portuguese version of the CAPD developed in the present study exhibited technical and semantic equivalence to the original version. One term was added to question #5 in the final version, where the terms *agitado*/*inquieto* were used.

The assessment of the equivalence between the items in the original scale, synthesis of the Portuguese-translated versions, and synthesis of the back-translations enables us to assert that most items, in both the translation and back-translation, are similar to those in the original scale; thus, the Portuguese version was rated as only slightly modified. In our opinion, the fact that no term within any item entirely differed between the original and translated version is because of the instrument's simplicity, given that it contains practical terms and is written using simple words.

During the multidisciplinary expert panel assessment, whenever questions arose about the meaning of a given item, the appendix included in the original publication of the CAPD was checked.^(^^12^^)^ The main author of the CAPD was consulted for additional explanations to preserve the semantic characteristics of the original instrument.

No term was changed after the expert panel reviewed the instrument because the scale represented a literal translation with the same meanings used in Brazil. The main focus of the discussion was the translation of the word "restless," for which the translators suggested the synonyms *agitada* and *inquieta*. Based on these experts' opinions and the need to preserve the semantic characteristics of the original instrument, we decided to keep both terms (i.e., *agitada/inquieta*) in the question. These changes introduced following the suggestions made by the expert panel improved our understanding of the instrument's items.

The examiner did not report any problems related to uncertainties or difficulties with regard to interpretation hindering performance during pretesting. These findings corroborate the belief that the items are pertinent to Brazilian culture and assess the dimension intended by the original instrument.

Although no rigorous gold standard exists regarding translation and cross-cultural adaptation for investigators, some guidelines recommend three crucial steps for this type of study: forward and backward translation, a review by an expert panel, and pretesting.^(^^20^^,^^21^^)^ All of these steps were rigorously followed in the present study.

## CONCLUSIONS

The Cornell Assessment of Pediatric Delirium has now been translated into Brazilian Portuguese and cross-culturally adapted for Brazil; thus, it is ready for large-scale testing. Future studies are needed to assess its reproducibility and psychometric properties to make its use feasible for all Brazilian regions.
